# Gastrointestinal (GI) Amyloidosis Presenting As Chronic Diarrhea: A Diagnostic Dilemma

**DOI:** 10.7759/cureus.41291

**Published:** 2023-07-02

**Authors:** Daniyal Raza, Poojan Prajapati, Vatsa Bhavsar, Syed Musa Raza, Ioannis Papayannis

**Affiliations:** 1 Internal Medicine, Louisiana State University Health Shreveport, Shreveport, USA; 2 Internal Medicine, B.J. Medical College, Ahmedabad, IND; 3 Gastroenterology, Louisiana State University Health Shreveport, Shreveport, USA

**Keywords:** congo red, systemic amyloidosis, shock, chronic diarrhea, amyloidosis

## Abstract

This case report describes a 63-year-old male patient with a four-year history of chronic diarrhea. Extensive diagnostic investigations failed to reveal a cause. Subsequent upper and lower gastrointestinal (GI) endoscopic procedures revealed the presence of amyloidosis in the GI tract. The patient was referred for further evaluation, but unfortunately, he presented with hypotension and shock, and ultimately succumbed to systemic amyloidosis involving multiple organs. GI amyloidosis, although rare, should be considered in patients presenting with chronic diarrhea, unexplained weight loss, or GI bleeding. Early recognition and appropriate management are crucial for optimizing patient outcomes. Healthcare providers should maintain a high index of suspicion for GI amyloidosis to ensure timely intervention and improve patient care.

## Introduction

Amyloidosis is a collective term for the extracellular deposition of abnormally folded insoluble proteins which are resistant to digestion, either in a single organ or throughout the body [1]. This leads to disruption in the functioning of the affected organs and systems. Chronic inflammatory conditions lead to an upregulation in the production of the serum amyloid-associated protein (SAA), an acute-phase reactant. This increased production of SAA subsequently results in the deposition of amyloid-associated (AA) protein within different organs throughout the body [2]. The deposition of AA proteins within the gastrointestinal (GI) tract can result in GI amyloidosis, which manifests with a range of clinical symptoms, primarily including nausea, vomiting, diarrhea, anorexia, malabsorption, esophageal reflux, and varying degrees of upper and lower GI bleeding [3]. 

Chronic diarrhea can arise from various causes and is considered one of the most prevalent symptoms with an approximate prevalence of 5% in the United States [4]. GI amyloidosis is one of the less frequent causes which should be considered in adult patients presenting with chronic diarrhea in which careful evaluation of many differential diagnoses yields no definite cause. In this case report, we present a 63-year-old male patient who had been experiencing chronic diarrhea for a duration of four years. Extensive diagnostic investigations revealed the presence of amyloidosis affecting a significant portion of the GI tract. 

## Case presentation

**Figure 1:**
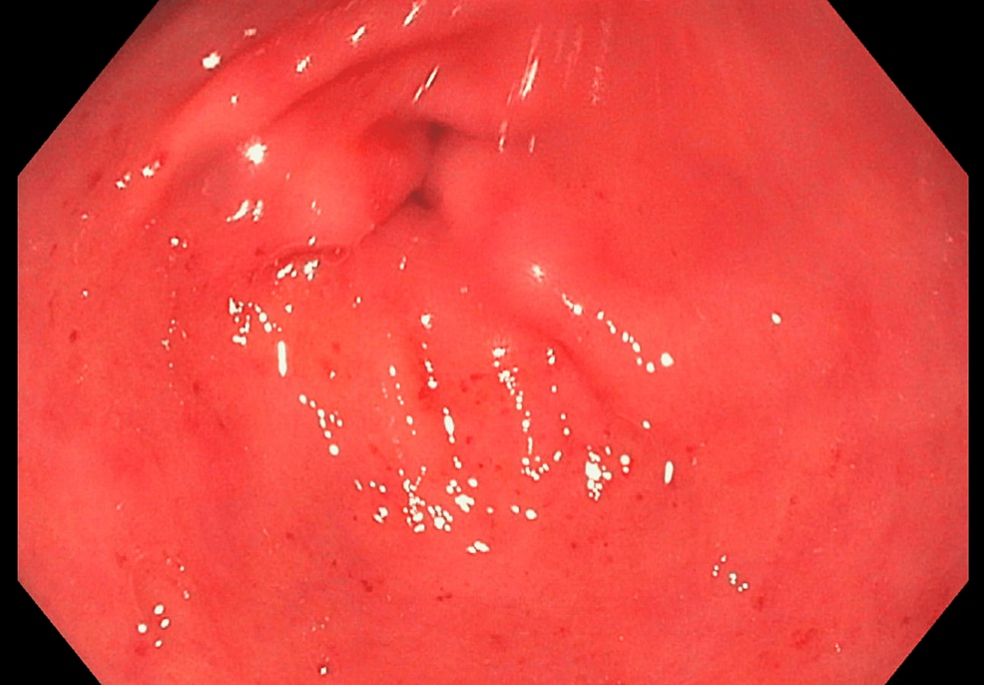
EGD revealed mild antral gastritis EGD: Upper gastrointestinal endoscopy

**Figure 2:**
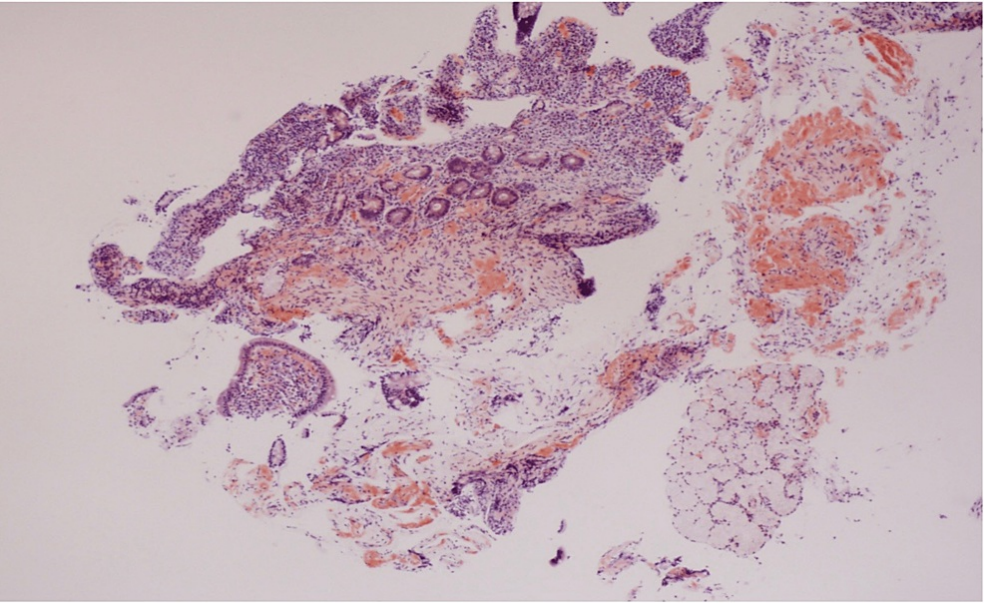
Photomicrographs showing duodenal mucosa with villous blunting, diffuse lymphoplasmacytic infiltrate within the lamina propria, and focal loss of Brunner glands. There is diffuse deposition of eosinophilic, amorphous material, which is positive for Congo red stain.

**Figure 3:**
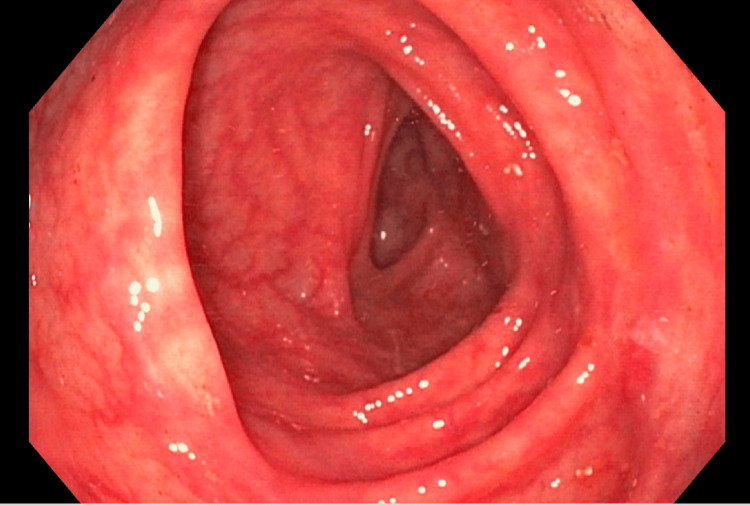
Transverse colon showed normal mucosa without any abnormalities or notable findings

A 63-year-old male with a past medical history of stroke, hypertension, and alcohol abuse (which he quit many years ago) presented to the gastroenterology clinic for evaluation of chronic diarrhea. The patient has been experiencing diarrhea for the past four years, with two-three daily occurrences. The stools have been consistently described as watery. Additionally, the patient reported an unintentional weight loss of seven pounds over the past few months. He denied having hematochezia, melena, fever, abdominal pain, nausea, vomiting, or any recent use of antibiotics. All stool studies yielded negative results, including the celiac panel, stool ova and parasites, Clostridium difficile, and Shigella toxin. Stool electrolyte levels were found to be within the normal range. The patient underwent both an upper GI endoscopy (EGD) and a colonoscopy. The EGD revealed the presence of mild antral gastritis (Figure 1). Biopsies were taken to rule out H. pylori and celiac disease. Pathology was negative for H. pylori infection. Biopsies of the duodenum (Figure 2) and stomach revealed diffuse deposition of eosinophilic, amorphous material, which stained positive for Congo red, consistent with amyloidosis. During the colonoscopy, the overall appearance of the colon was normal (as shown in Figure 3). However, a 4-mm sessile polyp with an adenomatous appearance was identified specifically in the sigmoid colon. This polyp was successfully removed using a cold snare technique. Multiple random biopsies were taken, and the pathology results revealed amyloidosis in the right colon, left colon, and sigmoid polyp. The patient was referred to hematology and oncology for further evaluation, given the amyloid deposits noted on pathology from EGD and colonoscopy. 

Before the patient could be seen by hematology and oncology, a month later, he visited the emergency department (ED) due to concerns about hypotension, despite being completely asymptomatic. He reported observing a pattern of consistently low blood pressure readings at home. For the past three days, his systolic blood pressure has ranged from 80s to 90s. The patient denied experiencing any other symptoms such as dizziness, lightheadedness, or chest pain. As per the patient, these were recent developments. The patient’s severe condition warranted admission to critical care to initiate vasopressors and perform further investigations. Unfortunately, he succumbed to shock, most likely cardiogenic. Subsequent autopsy findings revealed that the cause of death was systemic amyloidosis involving the heart, liver, and adrenal glands.

## Discussion

Amyloidosis can be classified based on various etiologies, including hereditary, ocular, central nervous system, and can occur as localized or systemic [3]. The most common types of amyloidosis include amyloid light-chain (AL) amyloidosis, AA amyloidosis, amyloid transport protein transthyretin (ATTR) amyloidosis, and dialysis-related amyloidosis (beta2M type). These classifications help in understanding the specific mechanisms and manifestations of amyloid deposition in different organs and tissues throughout the body. While localized involvement of the GI tract is rare, systemic amyloidosis is the predominant form found in the majority of cases, with GI tract involvement seen in approximately 50% of those cases [5]. Our patient likely had systemic amyloidosis, as the autopsy showed the involvement of multiple organs. GI amyloidosis should be considered as a potential differential diagnosis in patients presenting with symptoms of diarrhea, unexplained weight loss, or GI bleeding. These symptoms, in conjunction with the patient's medical history and the presence of disorders known to be associated with amyloidosis, should raise suspicion for GI involvement in amyloidosis. GI symptoms are prevalent in approximately 58.1% of patients with AA amyloidosis [6]. These symptoms can arise due to mucosal or neuromuscular infiltration by insoluble misfolded amyloid proteins and, in rare cases, extrinsic autonomic neuropathy [7,8]. AA amyloidosis involving the GI tract commonly presents with diarrhea and malabsorption, as granular amyloid deposition primarily occurs in the mucosa, resulting in a fine granular appearance, mucosal friability, and erosions [9]. In a study conducted by Tada et al., 100% of biopsy specimens from the second part of the duodenum, 95% from the stomach, 91% from the colorectum, and 72% from the esophagus exhibited amyloid deposition in amyloidosis patients with GI symptoms [10]. 

The diagnosis of GI amyloidosis typically involves an EDG and a tissue biopsy, with positive staining of amyloid by Congo red or the presence of amyloid fibrils on electron microscopy [3,11]. When stained with Congo red dye and examined under polarized light, amyloid exhibits a distinct and characteristic "apple green" birefringence. This optical property is a key feature used in the microscopic identification of amyloid deposits in tissues. Management of AA amyloidosis patients presenting with diarrhea primarily focuses on supportive care. In small intestinal bacterial overgrowth cases, empiric treatment with antibiotics (e.g., quinolones, doxycycline, metronidazole) may be beneficial. Glucocorticoids and octreotide have shown some efficacy in treating protein-losing enteropathy in certain patients [12,13]. Surgical intervention may be considered for severe complications such as perforation or GI hemorrhage [14,15]. 

## Conclusions

Our case highlights the diagnostic challenge of GI amyloidosis in patients presenting with chronic diarrhea. GI amyloidosis should be considered a potential differential diagnosis in patients with unexplained weight loss, GI bleeding, and persistent diarrhea, especially when other common causes have been ruled out. Diagnosis typically involves endoscopy and biopsy, with positive staining by Congo red or pathognomonic findings on electron microscopy. Early suspicion, prompt diagnosis, and appropriate management can significantly impact patients' outcomes and improve prognosis.
